# Crystal structures of bis­[4-(di­methyl­amino)­pyridinium] tetra­kis­(thio­cyanato-κ*N*)manganate(II) and tris­[4-(di­methyl­amino)­pyridinium] penta­kis(thio­cyanato-κ*N*)manganate(II)

**DOI:** 10.1107/S2056989017017510

**Published:** 2018-01-01

**Authors:** Tristan Neumann, Inke Jess, Christian Näther

**Affiliations:** a Institut für Anorganische Chemie, Christian-Albrechts-Universität Kiel, Max-Eyth Strasse 2, D-24118 Kiel, Germany

**Keywords:** crystal structure, discrete complexes, thio­cyanato­manganate(II), fivefold coordination, fourfold coordination, 4-(di­methyl­amino)­pyridine, hydrogen bonding

## Abstract

The crystal structures of the title salts, (C_7_H_11_N_2_)_2_[Mn(NCS)_4_] (**1**) and (C_7_H_11_N_2_)_3_[Mn(NCS)_5_] (**2**), consist of manganese(II) cations that are tetra­hedrally (**1**) or trigonal–bipyramidally (**2**) coordinated to four or five terminal N-bonded thio­cyanate ligands, respectively, into discrete anionic complexes. The negative charge is compensated by two (**1**) or three (**2**) 4-(di­methyl­amino)­pyridinium cations, which are protonated at the pyridine N atom. The asymmetric unit of compound **1** consists of one anionic complex and two 4-(di­methyl­amino)­pyridinium cations, whereas that of compound **2** consists of two anionic complexes and six 4-(di­methyl­amino)­pyridinium cations, all of them located in general positions. These complexes are linked by N—H⋯S, C—H⋯S and C—H⋯N hydrogen-bonding inter­actions between the 4-(di­methyl­amino)­pyridinium cations and the thio­cyanate ligands into three-dimensional network structures.

## Abstract 2

The crystal structures of the title salts consist of discrete anionic complexes, in which the Mn^II^ atom is either in a distorted tetra­hedral or a trigonal–bipyramidal coordination environment by terminal N-bonding thio­cyanate ligands. The complex anions are charge-balanced by two or three 4-(di­methyl­amino)­pyridinium cations.

## Chemical context   

**Figure 1:**
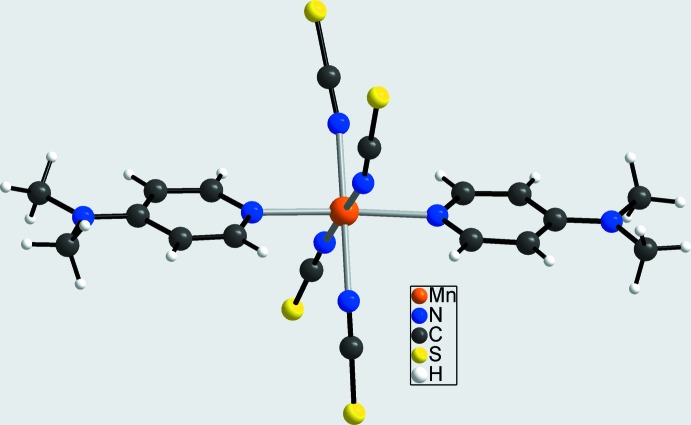
View of the Mn coordination in bis­[4-(di­methyl­amino)­pyridinium] bis­[4-(di­methyl­amino)­pyridine-κ*N*]tetra­kis­(thio­cyanato-κ*N*)manganate(II). Data taken from Wöhlert *et al.* (2012*b* ▸).

Thio­cyanate anions are versatile ligands that can be coordinated to metal cations in different ways. The most prominent coordin­ation modes include the terminal and the *μ*-1,3 coord­ination modes. The latter mode is of special importance for compounds showing cooperative magnetic phenomena (Palion-Gazda *et al.*, 2015 ▸; Massoud *et al.*, 2013 ▸; Mousavi *et al.*, 2012 ▸). In this context, we have reported a number of compounds based on *M*(NCS)_2_ moieties (*M* = Mn, Fe, Co and Ni) that show different magnetic properties including single-chain magnetism (Werner *et al.*, 2015*a* ▸,*b* ▸; Rams *et al.*, 2017*a* ▸,*b* ▸). In the majority of structures, the metal cations are linked by pairs of *μ*-1,3 bridging ligands into chains, but 2D networks are also realized in which the cations are linked by pairs and single anionic ligands into layers (Suckert *et al.*, 2016 ▸; Wöhlert *et al.*, 2012*a* ▸, 2013 ▸). In some cases, compounds comprising bridging anionic ligands need to be prepared by thermal decomposition of precursors that consist of discrete octa­hedral complexes with terminal N-bonded thio­cyanate anions. In this regard, we became inter­ested in mixed crystals based on Mn^II^ and Co^II^ atoms with the strong N-donor co-ligand 4-di­methyl­amino­pyridine that might be prepared by thermal decomposition of mixed crystals of the corresponding discrete precursor complexes. To prove mixed crystal formation, the X-ray diffraction powder pattern of all samples needs to be compared with physical mixtures with the same metal-to-metal ratio. We therefore attempted to prepare [Mn(NCS)_2_(4-(di­methyl­amino)­pyridine)_4_], but in all cases obtained only the salt-like crystals **1** and **2**, in which the Mn^II^ atom is solely coordinated by thio­cyanate ligands, either in a tetra­hedral (**1**) or trigonal–bipyramidal (**2**) configuration, and charge-balanced by 4-(di­methyl­amino)­pyridinium cations. The formation of these cations might be traced back to the fact that the neutral mol­ecule is a strong base because of the electron-donating di­methyl­amino substituent and therefore can easily be protonated. It should be mentioned that neither of the two compounds could be prepared in larger amounts as a pure crystalline phase, because mixtures were always obtained. However, both compounds are of inter­est from a structural point of view, because negatively charged manganate complexes with a fivefold coordination by thio­cyanate ligands are scarce. Moreover, a manganate(II) complex with 4-di­methyl­amino­pyridine has already been reported in the literature (Wöhlert *et al.*, 2012*b* ▸; Fig. 1 ▸). In the structure of this compound, the Mn^II^ atom is octa­hedrally coordinated to four terminal N-bonded thio­cyanate anions and two neutral 4-(di­methyl­amino)­pyridine ligands, and the twofold negative charge is compensated by two 4-(di­methyl­amino)­pyridinium cations. Therefore, the crystal structures of the title compounds **1** and **2** supplement the coordination polyhedra realized for thio­cyanato­manganate(II) complexes with 4-(di­methyl­amino)­pyridinium as counter-cationic species.
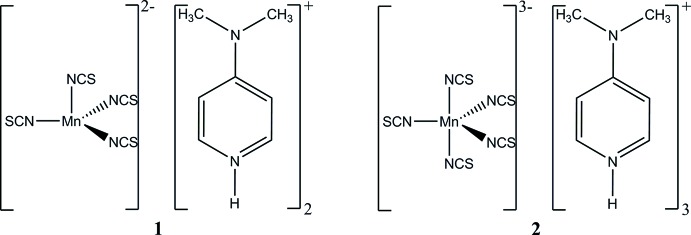


## Structural commentary   

**Figure 2:**
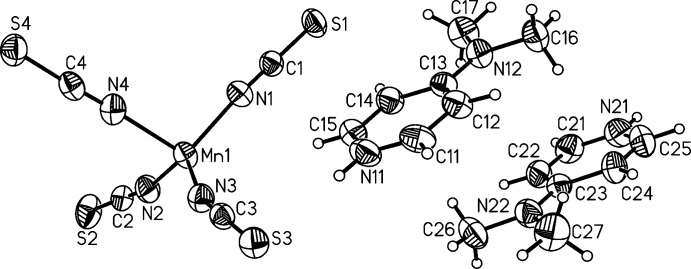
View of the asymmetric unit of **1**, with atomic labelling and displacement ellipsoids drawn at the 50% probability level.

**Figure 3:**
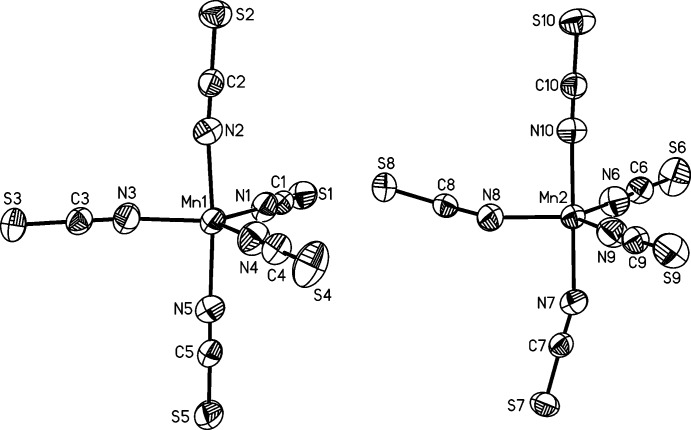
View of the Mn coordination in **2**, with atomic labelling and displacement ellipsoids drawn at the 50% probability level.

In the crystal structure of compound **1**, the Mn^II^ atom is surrounded by four terminal N-bonded thio­cyanate ligands within a considerably distorted tetra­hedral coordination sphere. The N—Mn—N bond angles vary from 93.83 (7)° to 123.57 (7)° (Fig. 2 ▸ and Table 1 ▸). The asymmetric unit of **1** comprises two cations and one complex anion. In contrast, the asymmetric unit of compound **2** comprises six cations and two anionic complexes, and the two Mn^II^ atoms in **2** are fivefold coordinated to the thio­cyanato anions. The resulting coord­in­ation polyhedra around the two central metal atoms can be described as distorted trigonal bipyramids (Fig. 3 ▸ and Table 2 ▸). This is supported by calculation of the structural parameter *τ*_5_ (Addison *et al.*, 1984 ▸), which leads to a value of 0.85 for Mn1 and of 0.93 for Mn2 (ideal value for a trigonal–bipyramidal coordination is 1, that of an ideal square-pyramidal coordination is 0). The Mn—N bond lengths in both independent complexes are comparable, but in both of them the distances to the thio­cyanate N atom in axial positions are significantly elongated, which might be the result of steric effects between the anionic ligands in the equatorial position (Tables 1 ▸ and 2 ▸). In the structure of **1**, three Mn—N bond lengths are similar, whereas the fourth is significantly elongated by about 0.07 Å (Table 1 ▸). When comparing the Mn—N bond lengths of **1** and **2** with those of bis­(4-(di­methyl­amino)­pyridinium) [bis­(4-(di­methyl­amino)-pyridine-κ*N*)tetra­kis­(thio­cyanato-κ*N*)manganate(II)] (Wöhlert *et al.*, 2012*b* ▸), it becomes obvious that they increase with increasing coordination number. The negative charge of the anionic complexes in compounds **1** and **2** is compensated by two or six, respectively, crystallographically independent 4-(di­methyl­amino)­pyridinium cations that are located in general positions (Figs. 2 ▸ and 4 ▸).

## Supra­molecular features   

**Figure 5:**
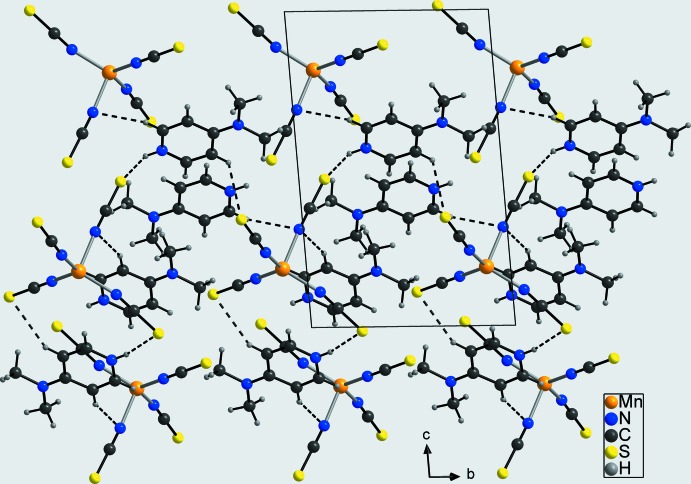
Crystal structure of compound **1** in a view along the *a* axis. Inter­molecular hydrogen bonding is shown as dashed lines.

**Figure 6:**
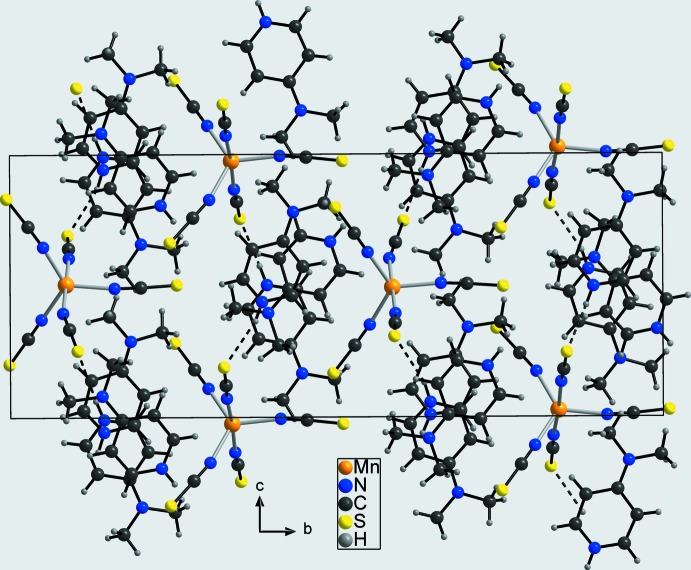
Crystal structure of compound **2** in a view along the *a* axis. Inter­molecular hydrogen bonding is shown as dashed lines. The polar character of this structure is emphasized by the same orientation of the complex anions relative to the *b* axis.

In the crystal structure of **1**, the negatively charged tetra­kis­(thio­cyanato)­manganese(II) complex mol­ecules are linked to the 4-(di­methyl­amino)­pyridinium cations by inter­molecular N—H⋯S and C—H⋯S hydrogen bonding between the pyridinium N—H group and C—H hydrogen atoms, and the thio­cyanate S atoms into a three-dimensional network (Fig. 5 ▸ and Table 3 ▸). There are two additional C—H⋯N contacts between the pyridinium C—H hydrogen atoms and the thio­cyanate N atom N4, which is exactly the N atom of the ligand that shows the elongated Mn—N bond length. In the crystal structure of **2**, inter­molecular N—H⋯S, C—H⋯S and C—H⋯ N hydrogen bonding between the thio­cyanate anions of the anionic complexes and the 4-(di­methyl­amino)­pyridinium cations is also observed, leading likewise to a three-dimensional hydrogen-bonded network (Fig. 6 ▸ and Table 4 ▸). The 4-(di­methyl­amino)­pyridinium cations are stacked along the *a* axis into columns, but are slightly shifted one to the other within these columns. More importantly, the two penta­kis(thio­cyanato)­manganese(II) complexes point in the same direction relative to the crystallographic *b* axis, from which the polar and non-centrosymmetric arrangement becomes obvious (Fig. 6 ▸).

## Database survey   

There are only two Mn^II^ thio­cyanate coordination polymers with 4-(di­methyl­amino)­pyridine reported in the Cambridge Structural Database (Version 5.38; Groom *et al.*, 2016 ▸). They include the bis­(4-(di­methyl­amino)­pyridinium) [bis­(4-(di­methyl­amino)­pyridine-κ*N*)tetra­kis­(thio­cyanato-κ*N*)mang­anate(II)] mentioned above (Wöhlert *et al.*, 2012*b* ▸) and the discrete complex bis­[4-(di­methyl­amino)­pyridine-κ*N*]bis(methanol-κ*O*)bis(thio­cyanato-κ*N*)manganese(II), in which the Mn^II^ cations are octa­hedrally coordinated to two terminal N-bonding thio­cyanate anions, two 4-(di­methyl­amino)­pyridine ligands and two methanol mol­ecules (Suckert *et al.*, 2015 ▸). There are a few compounds reported that are built up of discrete anionic manganate(II) complexes, in which the Mn^II^ atoms are in an octa­hedral coordination by six terminal N-bonding thio­cyanate ligands with different charge-compensating cations. They include tetra­kis­(tetra­methyl­phospho­nium) [hexa­kis­(thio­cyanato)­manganese(II)] (Li *et al.*, 2015 ▸), tetra­kis­(tetra­methyl­ammonium) [hexa­kis­(thio­cyanato)­mang­anese(II)] (Savard & Leznoff, 2013 ▸) and tetra­kis­(tris­(amino­eth­yl)amine)(thio­cyanato)­copper(II) [hexa­kis­(thio­cyanato)­manganese(II)] (Bose *et al.*, 2006 ▸). Similar compounds with five thio­cyanate anions coordinating to Mn^II^ are also known, but only a few have been reported (Matoga *et al.*, 2015 ▸; Savard & Leznoff, 2013 ▸; Hill *et al.*, 2008 ▸). Finally, some discrete Mn^II^ complexes with a fourfold thio­cyanate coordination are also known, such as in the salt bis­(tetra­phenyl­phospho­nium) [tetra­kis­(thio­cyanato)­manganese(II)] (Kushch *et al.*, 2014 ▸).

## Synthesis and crystallization   

MnSO_4_·H_2_O was obtained from Merck and Ba(NCS)_2_·3H_2_O from Alfa Aesar. Equimolar amounts of both compounds were reacted in water. The resulting white precipitate of BaSO_4_ was filtered off, and the filtrate was evaporated until complete dryness. The purity of the white residue of Mn(NCS)_2_ was checked by X-ray powder diffraction (XRPD) and thermogravimetry. For the synthesis of complex **1**, Mn(NCS)_2_ (1.0 mmol, 170 mg) was reacted with 4-(di­methyl­amino)­pyridine (0.5 mmol, 61.0 mg) in 1.0 ml of water. The precipitate was filtered off and the filtrate was allowed to stand under ambient conditions. After a few days, single crystals suitable for single-crystal X-ray diffraction had formed. For the synthesis of complex **2**, Mn(NCS)_2_ (1.0 mmol, 170 mg) was reacted with 4-(di­methyl­amino)­pyridine (1.0 mmol, 122 mg) in 4.0 ml of water. Single crystals formed from the filtrate at room temperature in a closed test tube after a few days. XRPD measurements proved that mixtures were always obtained, sometimes consisting of compound **1** and **2** or one of these compounds contaminated with additional crystalline phases.

## Refinement   

Crystal data, data collection and structure refinement details are summarized in Table 5 ▸. The C—H and N—H hydrogen atoms were initially located in difference maps but were finally positioned with idealized geometry (methyl H atoms allowed to rotate but not to tip) and were refined with fixed isotropic displacement parameters *U*_iso_(H) = 1.2*U*_eq_(C, N) for aromatic and *U*_iso_(H) = 1.5*U*_eq_(C) for methyl H atoms. For **2**, the Flack (1983 ▸) parameter did not refine to zero within the estimated standard deviation. Therefore, a twin refinement was performed leading to a BASF parameter of 0.028 (18). However, the non-centrosymmetric and polar arrangement is clearly seen in Fig. 6 ▸.

## Supplementary Material

Crystal structure: contains datablock(s) Compound1, Compound2. DOI: 10.1107/S2056989017017510/wm5426sup1.cif

Structure factors: contains datablock(s) Compound1. DOI: 10.1107/S2056989017017510/wm5426Compound1sup2.hkl

Structure factors: contains datablock(s) Compound2. DOI: 10.1107/S2056989017017510/wm5426Compound2sup3.hkl

CCDC references: 1589470, 1589469

Additional supporting information:  crystallographic information; 3D view; checkCIF report

## Figures and Tables

**Figure 4:**
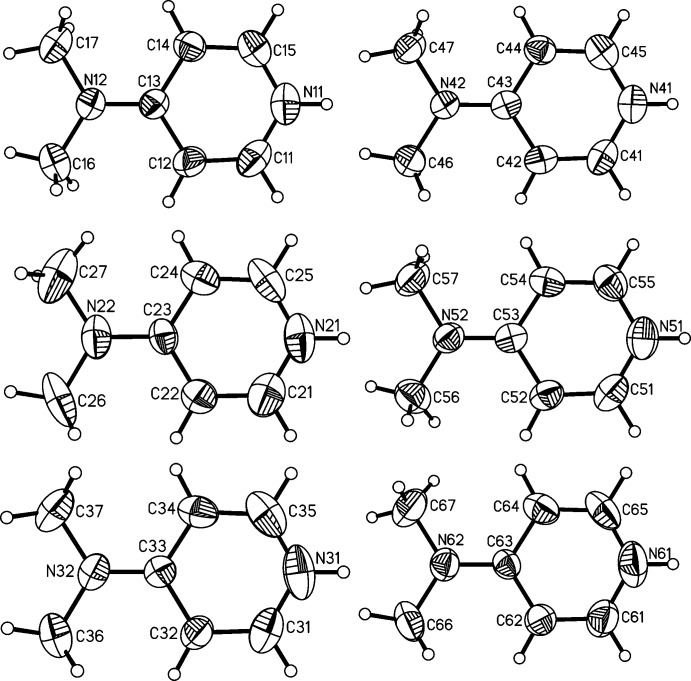
View of the six crystallographically independent 4-(di­methyl­amino)­pyridinium cations in **2**, with atomic labelling and displacement ellipsoids drawn at the 50% probability level.

**Table 1:** Selected geometric parameters (Å, °) for compound **1** [Chem scheme1]

Mn1—N1	2.0495 (17)	Mn1—N3	2.0810 (16)
Mn1—N2	2.0600 (16)	Mn1—N4	2.1336 (17)
			
N1—Mn1—N2	118.35 (7)	N1—Mn1—N4	101.27 (7)
N1—Mn1—N3	112.93 (6)	N2—Mn1—N4	93.83 (7)
N2—Mn1—N3	123.57 (7)	N3—Mn1—N4	97.99 (6)

**Table 2:** Selected geometric parameters (Å, °) for compound **2** [Chem scheme1]

Mn1—N4	2.099 (4)	Mn2—N6	2.100 (5)
Mn1—N1	2.104 (4)	Mn2—N8	2.100 (4)
Mn1—N3	2.128 (4)	Mn2—N9	2.103 (4)
Mn1—N2	2.198 (5)	Mn2—N10	2.205 (4)
Mn1—N5	2.205 (4)	Mn2—N7	2.217 (4)
			
N4—Mn1—N1	115.03 (17)	N6—Mn2—N8	121.35 (19)
N4—Mn1—N3	123.99 (19)	N6—Mn2—N9	121.77 (19)
N1—Mn1—N3	120.97 (18)	N8—Mn2—N9	116.88 (18)
N4—Mn1—N2	91.71 (19)	N6—Mn2—N10	90.22 (18)
N1—Mn1—N2	91.61 (18)	N8—Mn2—N10	89.77 (17)
N3—Mn1—N2	86.71 (17)	N9—Mn2—N10	89.23 (18)
N4—Mn1—N5	90.38 (17)	N6—Mn2—N7	92.53 (18)
N1—Mn1—N5	91.55 (17)	N8—Mn2—N7	90.07 (16)
N3—Mn1—N5	88.42 (17)	N9—Mn2—N7	88.06 (17)
N2—Mn1—N5	175.07 (16)	N10—Mn2—N7	176.88 (18)

**Table 3:** Hydrogen-bond geometry (Å, °) for **1** [Chem scheme1]

*D*—H⋯*A*	*D*—H	H⋯*A*	*D*⋯*A*	*D*—H⋯*A*
N11—H11*A*⋯S3	0.88	2.45	3.3129 (18)	166
C12—H12⋯N4^i^	0.95	2.67	3.548 (2)	155
C14—H14⋯S1	0.95	2.94	3.788 (2)	149
N21—H21*A*⋯S4^ii^	0.88	2.51	3.2771 (19)	147
C21—H21⋯N4^iii^	0.95	2.64	3.440 (3)	142
C24—H24⋯S2^iv^	0.95	2.85	3.548 (2)	131

Symmetry codes: (i) 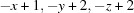; (ii) 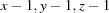; (iii) 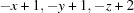; (iv) 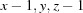.

**Table 4:** Hydrogen-bond geometry (Å, °) for **2** [Chem scheme1]

*D*—H⋯*A*	*D*—H	H⋯*A*	*D*⋯*A*	*D*—H⋯*A*
N11—H11*A*⋯S2	0.88	2.37	3.224 (4)	163
C11—H11⋯S9^i^	0.95	3.02	3.945 (5)	166
C15—H15⋯S8^ii^	0.95	2.86	3.728 (5)	153
C16—H16*B*⋯S9^iii^	0.98	3.02	3.954 (6)	160
C17—H17*B*⋯S9^iii^	0.98	2.96	3.930 (6)	170
N21—H21*A*⋯S1^iv^	0.88	2.82	3.520 (5)	138
N21—H21*A*⋯S7	0.88	2.81	3.485 (6)	134
C25—H25⋯N1^iv^	0.95	2.62	3.567 (7)	175
C26—H26*B*⋯N5	0.98	2.58	3.500 (7)	157
N31—H31*A*⋯S10	0.88	2.41	3.266 (5)	164
C31—H31⋯S3^v^	0.95	2.99	3.838 (6)	150
C35—H35⋯S1^vi^	0.95	2.96	3.512 (6)	118
C36—H36*B*⋯S3^vii^	0.98	2.99	3.868 (6)	149
N41—H41*A*⋯S5	0.88	2.45	3.302 (4)	163
C41—H41⋯S6	0.95	2.94	3.804 (5)	152
C45—H45⋯S8^viii^	0.95	2.88	3.445 (5)	119
C47—H47*C*⋯S2^ix^	0.98	2.98	3.717 (5)	133
N51—H51*A*⋯S7	0.88	2.43	3.288 (5)	163
C51—H51⋯S4^x^	0.95	2.99	3.931 (5)	169
C55—H55⋯S1^iv^	0.95	2.93	3.746 (5)	145
C57—H57*C*⋯N7^iv^	0.98	2.69	3.539 (7)	146
N61—H61*A*⋯S8^iv^	0.88	2.78	3.507 (5)	141
C65—H65⋯N8^iv^	0.95	2.66	3.513 (7)	150
C66—H66*A*⋯S4^x^	0.98	2.92	3.767 (6)	145

Symmetry codes: (i) 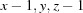; (ii) 
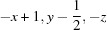
; (iii) 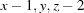; (iv) 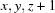; (v) 
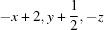
; (vi) 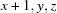; (vii) 
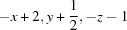
; (viii) 
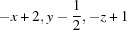
; (ix) 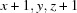; (x) 
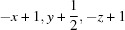
.

**Table 5:** Experimental details

	**1**	**2**
Crystal data		
Chemical formula	(C_7_H_11_N_2_)_2_[Mn(NCS)_4_]	(C_7_H_11_N_2_)_3_[Mn(NCS)_5_]
*M*_r_	533.61	714.87
Crystal system, space group	Triclinic, *P* 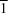	Monoclinic, *P*2_1_
Temperature (K)	170	170
*a*, *b*, *c* (Å)	8.5079 (4), 10.4356 (5), 14.9899 (7)	10.8320 (2), 28.1610 (5), 11.3392 (2)
α, β, γ (°)	93.748 (4), 90.464 (4), 112.585 (3)	90, 90.098 (1), 90
*V* (Å^3^)	1225.39 (10)	3458.90 (11)
*Z*	2	4
Radiation type	Mo *K*α	Mo *K*α
μ (mm^−1^)	0.90	0.72
Crystal size (mm)	0.22 × 0.12 × 0.06	0.25 × 0.18 × 0.10

Data collection		
Diffractometer	Stoe IPDS2	Stoe IPDS2
Absorption correction	Numerical (*X-SHAPE* and *X-RED32*; Stoe & Cie, 2008 ▸)	Numerical (*X-SHAPE* and *X-RED32*; Stoe & Cie, 2008 ▸)
*T*_min_, *T*_max_	0.593, 0.915	0.789, 0.915
No. of measured, independent and observed [*I* > 2σ(*I*)] reflections	17932, 5342, 4633	45580, 13568, 12684
*R*_int_	0.046	0.037
(sin θ/λ)_max_ (Å^−1^)	0.639	0.617

Refinement		
*R*[*F* ^2^ > 2σ(*F* ^2^)], *wR*(*F* ^2^), *S*	0.035, 0.098, 1.06	0.039, 0.099, 1.05
No. of reflections	5342	13568
No. of parameters	285	789
No. of restraints	0	1
H-atom treatment	H-atom parameters constrained	H-atom parameters constrained
Δρ_max_, Δρ_min_ (e Å^−3^)	0.31, −0.53	0.33, −0.24
Absolute structure	–	Refined as an inversion twin
Absolute structure parameter	–	0.028 (18)

Computer programs: *X-AREA* (Stoe & Cie, 2008 ▸), *XP* in *SHELXTL* and *SHELXS97* (Sheldrick, 2008 ▸), *SHELXL2014* (Sheldrick, 2015 ▸), *DIAMOND* (Brandenburg, 1999 ▸) and *publCIF* (Westrip, 2010 ▸).

## supplementary crystallographic information

### Bis[4-(dimethylamino)pyridinium] tetrakis(thiocyanato-κN)manganate(II) (Compound1) Crystal data

**Table:**

(C_7_H_11_N_2_)_2_[Mn(NCS)_4_]	*Z* = 2
*M**_r_* = 533.61	*F*(000) = 550
Triclinic, *P*1	*D*_x_ = 1.446 Mg m^−^^3^
*a* = 8.5079 (4) Å	Mo *K*α radiation, λ = 0.71073 Å
*b* = 10.4356 (5) Å	Cell parameters from 17932 reflections
*c* = 14.9899 (7) Å	θ = 2.1–27.0°
α = 93.748 (4)°	µ = 0.90 mm^−^^1^
β = 90.464 (4)°	*T* = 170 K
γ = 112.585 (3)°	Block, colorless
*V* = 1225.39 (10) Å^3^	0.22 × 0.12 × 0.06 mm

### Bis[4-(dimethylamino)pyridinium] tetrakis(thiocyanato-κN)manganate(II) (Compound1) Data collection

**Table:**

Stoe IPDS-2 diffractometer	4633 reflections with *I* > 2σ(*I*)
ω scans	*R*_int_ = 0.046
Absorption correction: numerical (X-SHAPE and X-RED32; Stoe & Cie, 2008)	θ_max_ = 27.0°, θ_min_ = 2.1°
*T*_min_ = 0.593, *T*_max_ = 0.915	*h* = −10→10
17932 measured reflections	*k* = −13→13
5342 independent reflections	*l* = −19→19

### Bis[4-(dimethylamino)pyridinium] tetrakis(thiocyanato-κN)manganate(II) (Compound1) Refinement

**Table:**

Refinement on *F*^2^	Hydrogen site location: inferred from neighbouring sites
Least-squares matrix: full	H-atom parameters constrained
*R*[*F*^2^ > 2σ(*F*^2^)] = 0.035	*w* = 1/[σ^2^(*F*_o_^2^) + (0.0601*P*)^2^ + 0.0944*P*] where *P* = (*F*_o_^2^ + 2*F*_c_^2^)/3
*wR*(*F*^2^) = 0.098	(Δ/σ)_max_ = 0.003
*S* = 1.06	Δρ_max_ = 0.31 e Å^−^^3^
5342 reflections	Δρ_min_ = −0.53 e Å^−^^3^
285 parameters	Extinction correction: SHELXL2014 (Sheldrick, 2015), Fc^*^=kFc[1+0.001xFc^2^λ^3^/sin(2θ)]^-1/4^
0 restraints	Extinction coefficient: 0.017 (3)

### Bis[4-(dimethylamino)pyridinium] tetrakis(thiocyanato-κN)manganate(II) (Compound1) Special details

**Table:**

Geometry. All esds (except the esd in the dihedral angle between two l.s. planes) are estimated using the full covariance matrix. The cell esds are taken into account individually in the estimation of esds in distances, angles and torsion angles; correlations between esds in cell parameters are only used when they are defined by crystal symmetry. An approximate (isotropic) treatment of cell esds is used for estimating esds involving l.s. planes.

### Bis[4-(dimethylamino)pyridinium] tetrakis(thiocyanato-κN)manganate(II) (Compound1) Fractional atomic coordinates and isotropic or equivalent isotropic displacement parameters (Å^2^)

**Table:**

	*x*	*y*	*z*	*U*_iso_*/*U*_eq_	
Mn1	0.90952 (3)	0.88265 (3)	1.18620 (2)	0.03782 (10)	
N1	0.6639 (2)	0.74716 (16)	1.15632 (11)	0.0426 (3)	
C1	0.5334 (2)	0.65751 (19)	1.13644 (12)	0.0388 (4)	
S1	0.35333 (7)	0.53200 (6)	1.10859 (4)	0.05316 (15)	
N2	1.0772 (2)	0.80600 (17)	1.23903 (11)	0.0451 (4)	
C2	1.1541 (2)	0.76211 (19)	1.28340 (12)	0.0402 (4)	
S2	1.26376 (9)	0.70138 (7)	1.34362 (4)	0.06153 (18)	
N3	0.9843 (2)	1.05670 (15)	1.11196 (10)	0.0415 (3)	
C3	0.9911 (2)	1.13584 (17)	1.05982 (12)	0.0356 (3)	
S3	0.99897 (6)	1.24678 (4)	0.98674 (3)	0.04241 (13)	
N4	0.8810 (2)	0.98070 (17)	1.31098 (11)	0.0470 (4)	
C4	0.8722 (2)	1.04124 (18)	1.37713 (13)	0.0400 (4)	
S4	0.85130 (7)	1.12578 (5)	1.46840 (3)	0.05017 (14)	
N11	0.6345 (2)	1.00661 (18)	0.90468 (12)	0.0496 (4)	
H11A	0.7252	1.0809	0.9219	0.060*	
C11	0.5288 (3)	1.0139 (2)	0.84009 (15)	0.0488 (5)	
H11	0.5525	1.0994	0.8137	0.059*	
C12	0.3886 (2)	0.90088 (19)	0.81197 (14)	0.0427 (4)	
H12	0.3155	0.9081	0.7660	0.051*	
C13	0.3498 (2)	0.77195 (18)	0.85038 (12)	0.0370 (4)	
C14	0.4655 (2)	0.7706 (2)	0.91879 (13)	0.0441 (4)	
H14	0.4458	0.6875	0.9474	0.053*	
C15	0.6036 (3)	0.8871 (2)	0.94344 (14)	0.0498 (5)	
H15	0.6802	0.8844	0.9890	0.060*	
N12	0.2129 (2)	0.65910 (15)	0.82409 (11)	0.0418 (3)	
C16	0.0935 (3)	0.6621 (2)	0.75498 (14)	0.0514 (5)	
H16A	0.0405	0.7267	0.7753	0.077*	
H16B	0.0053	0.5686	0.7429	0.077*	
H16C	0.1545	0.6930	0.7002	0.077*	
C17	0.1683 (3)	0.5301 (2)	0.86837 (16)	0.0534 (5)	
H17A	0.2515	0.4884	0.8543	0.080*	
H17B	0.0544	0.4651	0.8473	0.080*	
H17C	0.1691	0.5500	0.9332	0.080*	
N21	0.2097 (2)	0.34211 (19)	0.56590 (13)	0.0546 (4)	
H21A	0.1323	0.2595	0.5488	0.066*	
C21	0.3276 (3)	0.3541 (2)	0.62914 (15)	0.0523 (5)	
H21	0.3262	0.2730	0.6550	0.063*	
C22	0.4485 (3)	0.4792 (2)	0.65702 (13)	0.0465 (4)	
H22	0.5300	0.4850	0.7024	0.056*	
C23	0.4545 (2)	0.60157 (19)	0.61898 (12)	0.0389 (4)	
C24	0.3267 (3)	0.5837 (2)	0.55258 (14)	0.0483 (4)	
H24	0.3240	0.6623	0.5250	0.058*	
C25	0.2088 (3)	0.4556 (2)	0.52819 (15)	0.0550 (5)	
H25	0.1237	0.4454	0.4837	0.066*	
N22	0.5730 (2)	0.72629 (17)	0.64412 (12)	0.0487 (4)	
C26	0.7061 (3)	0.7430 (3)	0.71058 (18)	0.0656 (6)	
H26A	0.6552	0.6923	0.7626	0.098*	
H26B	0.7660	0.8420	0.7292	0.098*	
H26C	0.7870	0.7061	0.6847	0.098*	
C27	0.5877 (4)	0.8505 (2)	0.59983 (18)	0.0678 (7)	
H27A	0.6025	0.8358	0.5357	0.102*	
H27B	0.6864	0.9301	0.6255	0.102*	
H27C	0.4842	0.8691	0.6085	0.102*	

### Bis[4-(dimethylamino)pyridinium] tetrakis(thiocyanato-κN)manganate(II) (Compound1) Atomic displacement parameters (Å^2^)

**Table:**

	*U*^11^	*U*^22^	*U*^33^	*U*^12^	*U*^13^	*U*^23^
Mn1	0.03611 (17)	0.03304 (15)	0.04112 (17)	0.00946 (11)	−0.00592 (11)	0.00585 (11)
N1	0.0365 (8)	0.0408 (8)	0.0474 (9)	0.0115 (7)	−0.0022 (7)	0.0034 (6)
C1	0.0397 (10)	0.0410 (9)	0.0364 (9)	0.0158 (8)	−0.0009 (7)	0.0064 (7)
S1	0.0411 (3)	0.0541 (3)	0.0484 (3)	0.0003 (2)	−0.0078 (2)	0.0083 (2)
N2	0.0444 (9)	0.0483 (9)	0.0439 (8)	0.0191 (7)	−0.0069 (7)	0.0058 (7)
C2	0.0434 (10)	0.0411 (9)	0.0353 (9)	0.0162 (8)	−0.0011 (7)	−0.0007 (7)
S2	0.0784 (4)	0.0761 (4)	0.0476 (3)	0.0500 (3)	−0.0149 (3)	0.0008 (3)
N3	0.0451 (9)	0.0358 (7)	0.0408 (8)	0.0126 (6)	−0.0011 (7)	0.0028 (6)
C3	0.0335 (9)	0.0313 (8)	0.0382 (9)	0.0088 (6)	−0.0014 (7)	−0.0010 (7)
S3	0.0471 (3)	0.0316 (2)	0.0448 (3)	0.01048 (18)	−0.00020 (19)	0.00712 (17)
N4	0.0538 (10)	0.0439 (8)	0.0413 (9)	0.0171 (7)	−0.0050 (7)	0.0015 (7)
C4	0.0387 (9)	0.0323 (8)	0.0438 (10)	0.0070 (7)	−0.0065 (7)	0.0091 (7)
S4	0.0552 (3)	0.0454 (3)	0.0440 (3)	0.0139 (2)	−0.0023 (2)	−0.0027 (2)
N11	0.0346 (8)	0.0458 (9)	0.0599 (10)	0.0069 (7)	0.0021 (7)	−0.0013 (8)
C11	0.0402 (10)	0.0386 (9)	0.0672 (13)	0.0135 (8)	0.0091 (9)	0.0106 (9)
C12	0.0377 (10)	0.0396 (9)	0.0523 (11)	0.0153 (8)	0.0011 (8)	0.0114 (8)
C13	0.0351 (9)	0.0358 (8)	0.0412 (9)	0.0143 (7)	0.0033 (7)	0.0051 (7)
C14	0.0408 (10)	0.0472 (10)	0.0451 (10)	0.0165 (8)	0.0008 (8)	0.0107 (8)
C15	0.0396 (10)	0.0616 (12)	0.0461 (10)	0.0171 (9)	−0.0024 (8)	0.0052 (9)
N12	0.0430 (9)	0.0338 (7)	0.0458 (8)	0.0117 (6)	−0.0038 (7)	0.0043 (6)
C16	0.0472 (11)	0.0502 (11)	0.0503 (11)	0.0124 (9)	−0.0109 (9)	0.0003 (9)
C17	0.0568 (13)	0.0335 (9)	0.0645 (13)	0.0108 (8)	−0.0009 (10)	0.0085 (9)
N21	0.0515 (10)	0.0440 (9)	0.0579 (11)	0.0078 (8)	0.0016 (8)	−0.0020 (8)
C21	0.0605 (13)	0.0422 (10)	0.0512 (11)	0.0154 (9)	0.0049 (10)	0.0108 (8)
C22	0.0476 (11)	0.0489 (10)	0.0439 (10)	0.0185 (9)	−0.0011 (8)	0.0124 (8)
C23	0.0360 (9)	0.0418 (9)	0.0379 (9)	0.0132 (7)	0.0029 (7)	0.0063 (7)
C24	0.0536 (12)	0.0473 (10)	0.0469 (10)	0.0227 (9)	−0.0086 (9)	0.0039 (8)
C25	0.0521 (12)	0.0583 (12)	0.0535 (12)	0.0220 (10)	−0.0133 (10)	−0.0056 (10)
N22	0.0433 (9)	0.0448 (9)	0.0498 (9)	0.0073 (7)	−0.0026 (7)	0.0083 (7)
C26	0.0434 (12)	0.0719 (15)	0.0663 (15)	0.0060 (11)	−0.0124 (11)	0.0028 (12)
C27	0.0812 (17)	0.0412 (11)	0.0683 (15)	0.0083 (11)	0.0008 (13)	0.0123 (10)

### Bis[4-(dimethylamino)pyridinium] tetrakis(thiocyanato-κN)manganate(II) (Compound1) Geometric parameters (Å, º)

**Table:**

Mn1—N1	2.0495 (17)	C16—H16A	0.9800
Mn1—N2	2.0600 (16)	C16—H16B	0.9800
Mn1—N3	2.0810 (16)	C16—H16C	0.9800
Mn1—N4	2.1336 (17)	C17—H17A	0.9800
N1—C1	1.165 (2)	C17—H17B	0.9800
C1—S1	1.6169 (19)	C17—H17C	0.9800
N2—C2	1.159 (2)	N21—C21	1.339 (3)
C2—S2	1.6133 (19)	N21—C25	1.348 (3)
N3—C3	1.159 (2)	N21—H21A	0.8800
C3—S3	1.6286 (18)	C21—C22	1.351 (3)
N4—C4	1.159 (3)	C21—H21	0.9500
C4—S4	1.627 (2)	C22—C23	1.416 (3)
N11—C11	1.342 (3)	C22—H22	0.9500
N11—C15	1.344 (3)	C23—N22	1.332 (2)
N11—H11A	0.8800	C23—C24	1.418 (3)
C11—C12	1.356 (3)	C24—C25	1.351 (3)
C11—H11	0.9500	C24—H24	0.9500
C12—C13	1.419 (2)	C25—H25	0.9500
C12—H12	0.9500	N22—C26	1.454 (3)
C13—N12	1.333 (2)	N22—C27	1.458 (3)
C13—C14	1.420 (3)	C26—H26A	0.9800
C14—C15	1.354 (3)	C26—H26B	0.9800
C14—H14	0.9500	C26—H26C	0.9800
C15—H15	0.9500	C27—H27A	0.9800
N12—C16	1.455 (2)	C27—H27B	0.9800
N12—C17	1.458 (2)	C27—H27C	0.9800
			
N1—Mn1—N2	118.35 (7)	H16B—C16—H16C	109.5
N1—Mn1—N3	112.93 (6)	N12—C17—H17A	109.5
N2—Mn1—N3	123.57 (7)	N12—C17—H17B	109.5
N1—Mn1—N4	101.27 (7)	H17A—C17—H17B	109.5
N2—Mn1—N4	93.83 (7)	N12—C17—H17C	109.5
N3—Mn1—N4	97.99 (6)	H17A—C17—H17C	109.5
C1—N1—Mn1	171.10 (15)	H17B—C17—H17C	109.5
N1—C1—S1	179.42 (18)	C21—N21—C25	120.47 (18)
C2—N2—Mn1	166.86 (16)	C21—N21—H21A	119.8
N2—C2—S2	178.85 (18)	C25—N21—H21A	119.8
C3—N3—Mn1	164.22 (15)	N21—C21—C22	121.43 (19)
N3—C3—S3	179.51 (17)	N21—C21—H21	119.3
C4—N4—Mn1	175.99 (16)	C22—C21—H21	119.3
N4—C4—S4	177.36 (18)	C21—C22—C23	120.43 (19)
C11—N11—C15	120.80 (18)	C21—C22—H22	119.8
C11—N11—H11A	119.6	C23—C22—H22	119.8
C15—N11—H11A	119.6	N22—C23—C22	122.04 (17)
N11—C11—C12	120.89 (18)	N22—C23—C24	121.91 (17)
N11—C11—H11	119.6	C22—C23—C24	116.06 (18)
C12—C11—H11	119.6	C25—C24—C23	120.49 (18)
C11—C12—C13	120.75 (18)	C25—C24—H24	119.8
C11—C12—H12	119.6	C23—C24—H24	119.8
C13—C12—H12	119.6	N21—C25—C24	121.1 (2)
N12—C13—C12	122.07 (16)	N21—C25—H25	119.4
N12—C13—C14	122.04 (16)	C24—C25—H25	119.4
C12—C13—C14	115.89 (17)	C23—N22—C26	121.46 (18)
C15—C14—C13	120.35 (18)	C23—N22—C27	121.96 (18)
C15—C14—H14	119.8	C26—N22—C27	116.23 (19)
C13—C14—H14	119.8	N22—C26—H26A	109.5
N11—C15—C14	121.32 (19)	N22—C26—H26B	109.5
N11—C15—H15	119.3	H26A—C26—H26B	109.5
C14—C15—H15	119.3	N22—C26—H26C	109.5
C13—N12—C16	121.63 (16)	H26A—C26—H26C	109.5
C13—N12—C17	121.40 (16)	H26B—C26—H26C	109.5
C16—N12—C17	116.78 (16)	N22—C27—H27A	109.5
N12—C16—H16A	109.5	N22—C27—H27B	109.5
N12—C16—H16B	109.5	H27A—C27—H27B	109.5
H16A—C16—H16B	109.5	N22—C27—H27C	109.5
N12—C16—H16C	109.5	H27A—C27—H27C	109.5
H16A—C16—H16C	109.5	H27B—C27—H27C	109.5

### Bis[4-(dimethylamino)pyridinium] tetrakis(thiocyanato-κN)manganate(II) (Compound1) Hydrogen-bond geometry (Å, º)

**Table:**

*D*—H···*A*	*D*—H	H···*A*	*D*···*A*	*D*—H···*A*
N11—H11*A*···S3	0.88	2.45	3.3129 (18)	166
C12—H12···N4^i^	0.95	2.67	3.548 (2)	155
C14—H14···S1	0.95	2.94	3.788 (2)	149
N21—H21*A*···S4^ii^	0.88	2.51	3.2771 (19)	147
C21—H21···N4^iii^	0.95	2.64	3.440 (3)	142
C24—H24···S2^iv^	0.95	2.85	3.548 (2)	131

Symmetry codes: (i) −*x*+1, −*y*+2, −*z*+2; (ii) *x*−1, *y*−1, *z*−1; (iii) −*x*+1, −*y*+1, −*z*+2; (iv) *x*−1, *y*, *z*−1.

### Tris[4-(dimethylamino)pyridinium] pentakis(thiocyanato-κN)manganate(II) (Compound2) Crystal data

**Table:**

(C_7_H_11_N_2_)_3_[Mn(NCS)_5_]	*F*(000) = 1484
*M**_r_* = 714.87	*D*_x_ = 1.373 Mg m^−^^3^
Monoclinic, *P*2_1_	Mo *K*α radiation, λ = 0.71073 Å
*a* = 10.8320 (2) Å	Cell parameters from 45580 reflections
*b* = 28.1610 (5) Å	θ = 1.5–26.0°
*c* = 11.3392 (2) Å	µ = 0.72 mm^−^^1^
β = 90.098 (1)°	*T* = 170 K
*V* = 3458.90 (11) Å^3^	Block, colorless
*Z* = 4	0.25 × 0.18 × 0.10 mm

### Tris[4-(dimethylamino)pyridinium] pentakis(thiocyanato-κN)manganate(II) (Compound2) Data collection

**Table:**

Stoe IPDS-2 diffractometer	12684 reflections with *I* > 2σ(*I*)
ω scans	*R*_int_ = 0.037
Absorption correction: numerical (X-SHAPE and X-RED32; Stoe & Cie, 2008)	θ_max_ = 26.0°, θ_min_ = 1.5°
*T*_min_ = 0.789, *T*_max_ = 0.915	*h* = −13→13
45580 measured reflections	*k* = −34→34
13568 independent reflections	*l* = −13→13

### Tris[4-(dimethylamino)pyridinium] pentakis(thiocyanato-κN)manganate(II) (Compound2) Refinement

**Table:**

Refinement on *F*^2^	H-atom parameters constrained
Least-squares matrix: full	*w* = 1/[σ^2^(*F*_o_^2^) + (0.0506*P*)^2^ + 1.5196*P*] where *P* = (*F*_o_^2^ + 2*F*_c_^2^)/3
*R*[*F*^2^ > 2σ(*F*^2^)] = 0.039	(Δ/σ)_max_ = 0.001
*wR*(*F*^2^) = 0.099	Δρ_max_ = 0.33 e Å^−^^3^
*S* = 1.05	Δρ_min_ = −0.24 e Å^−^^3^
13568 reflections	Extinction correction: SHELXL2014 (Sheldrick, 2015), Fc^*^=kFc[1+0.001xFc^2^λ^3^/sin(2θ)]^-1/4^
789 parameters	Extinction coefficient: 0.0053 (7)
1 restraint	Absolute structure: Refined as an inversion twin
Hydrogen site location: inferred from neighbouring sites	Absolute structure parameter: 0.028 (18)

### Tris[4-(dimethylamino)pyridinium] pentakis(thiocyanato-κN)manganate(II) (Compound2) Special details

**Table:**

Geometry. All esds (except the esd in the dihedral angle between two l.s. planes) are estimated using the full covariance matrix. The cell esds are taken into account individually in the estimation of esds in distances, angles and torsion angles; correlations between esds in cell parameters are only used when they are defined by crystal symmetry. An approximate (isotropic) treatment of cell esds is used for estimating esds involving l.s. planes.
Refinement. Refined as a 2-component inversion twin.

### Tris[4-(dimethylamino)pyridinium] pentakis(thiocyanato-κN)manganate(II) (Compound2) Fractional atomic coordinates and isotropic or equivalent isotropic displacement parameters (Å^2^)

**Table:**

	*x*	*y*	*z*	*U*_iso_*/*U*_eq_	
Mn1	0.45124 (7)	0.33957 (2)	−0.02498 (5)	0.04095 (16)	
N1	0.4568 (4)	0.41385 (15)	−0.0058 (3)	0.0519 (10)	
C1	0.4812 (4)	0.45416 (16)	−0.0112 (4)	0.0415 (9)	
S1	0.51506 (14)	0.50955 (5)	−0.02592 (13)	0.0588 (3)	
N2	0.2830 (4)	0.34375 (18)	−0.1331 (4)	0.0635 (12)	
C2	0.1876 (5)	0.34774 (16)	−0.1793 (4)	0.0455 (10)	
S2	0.05434 (12)	0.35258 (5)	−0.24442 (11)	0.0533 (3)	
N3	0.5353 (4)	0.30569 (17)	−0.1721 (4)	0.0583 (11)	
C3	0.5780 (4)	0.28033 (17)	−0.2411 (4)	0.0444 (10)	
S3	0.63768 (14)	0.24496 (6)	−0.33664 (12)	0.0636 (4)	
N4	0.3576 (4)	0.30326 (16)	0.1095 (4)	0.0561 (10)	
C4	0.3062 (5)	0.28207 (16)	0.1821 (4)	0.0462 (10)	
S4	0.23426 (17)	0.25235 (5)	0.28177 (12)	0.0688 (4)	
N5	0.6269 (4)	0.33145 (15)	0.0716 (3)	0.0504 (9)	
C5	0.7204 (5)	0.33024 (16)	0.1210 (4)	0.0435 (10)	
S5	0.85205 (14)	0.32725 (6)	0.19012 (13)	0.0694 (4)	
Mn2	0.98322 (6)	0.58569 (2)	0.49368 (6)	0.04102 (16)	
N6	0.8987 (5)	0.55087 (17)	0.3511 (4)	0.0623 (11)	
C6	0.8649 (5)	0.52786 (18)	0.2728 (4)	0.0507 (11)	
S6	0.8161 (2)	0.49483 (7)	0.16721 (14)	0.0859 (5)	
N7	0.8142 (4)	0.58111 (16)	0.6030 (4)	0.0520 (9)	
C7	0.7313 (4)	0.59084 (16)	0.6640 (4)	0.0428 (9)	
S7	0.61231 (12)	0.60318 (5)	0.74658 (11)	0.0538 (3)	
N8	0.9804 (4)	0.66003 (15)	0.5084 (4)	0.0522 (10)	
C8	1.0076 (4)	0.70025 (16)	0.5056 (4)	0.0409 (9)	
S8	1.04620 (13)	0.75529 (4)	0.49639 (13)	0.0574 (3)	
N9	1.0767 (4)	0.54807 (16)	0.6268 (4)	0.0556 (10)	
C9	1.1319 (4)	0.52764 (16)	0.6988 (4)	0.0428 (9)	
S9	1.20997 (14)	0.49886 (5)	0.79725 (12)	0.0594 (3)	
N10	1.1573 (4)	0.59021 (16)	0.3939 (4)	0.0566 (10)	
C10	1.2582 (4)	0.58970 (16)	0.3575 (4)	0.0447 (10)	
S10	1.39824 (12)	0.58848 (6)	0.30722 (11)	0.0585 (3)	
N11	0.1399 (4)	0.39353 (17)	−0.4969 (4)	0.0570 (11)	
H11A	0.1338	0.3825	−0.4246	0.068*	
C11	0.1660 (5)	0.4390 (2)	−0.5146 (5)	0.0585 (13)	
H11	0.1807	0.4587	−0.4480	0.070*	
C12	0.1727 (5)	0.45877 (17)	−0.6236 (4)	0.0506 (11)	
H12	0.1910	0.4915	−0.6327	0.061*	
C13	0.1516 (4)	0.42950 (16)	−0.7241 (4)	0.0409 (9)	
C14	0.1284 (4)	0.38056 (16)	−0.7017 (4)	0.0462 (10)	
H14	0.1169	0.3592	−0.7656	0.055*	
C15	0.1227 (5)	0.36442 (18)	−0.5888 (5)	0.0541 (12)	
H15	0.1062	0.3318	−0.5749	0.065*	
N12	0.1540 (4)	0.44690 (14)	−0.8332 (3)	0.0489 (9)	
C16	0.1790 (6)	0.4965 (2)	−0.8554 (5)	0.0652 (14)	
H16A	0.1216	0.5161	−0.8097	0.098*	
H16B	0.1684	0.5032	−0.9396	0.098*	
H16C	0.2639	0.5039	−0.8318	0.098*	
C17	0.1250 (6)	0.4171 (2)	−0.9341 (4)	0.0662 (15)	
H17A	0.1778	0.3888	−0.9330	0.099*	
H17B	0.1395	0.4349	−1.0070	0.099*	
H17C	0.0382	0.4074	−0.9304	0.099*	
N21	0.5064 (5)	0.4907 (2)	0.6673 (4)	0.0686 (13)	
H21A	0.5138	0.5111	0.7258	0.082*	
C21	0.5188 (6)	0.5055 (2)	0.5574 (6)	0.0695 (15)	
H21	0.5324	0.5383	0.5425	0.083*	
C22	0.5126 (5)	0.47491 (19)	0.4656 (5)	0.0566 (12)	
H22	0.5234	0.4861	0.3873	0.068*	
C23	0.4903 (4)	0.42656 (18)	0.4869 (4)	0.0432 (10)	
C24	0.4738 (5)	0.4129 (2)	0.6041 (4)	0.0556 (12)	
H24	0.4562	0.3808	0.6226	0.067*	
C25	0.4826 (5)	0.4452 (3)	0.6904 (4)	0.0663 (16)	
H25	0.4718	0.4354	0.7699	0.080*	
N22	0.4861 (4)	0.39534 (16)	0.3968 (4)	0.0597 (11)	
C26	0.4996 (7)	0.4112 (3)	0.2744 (5)	0.085 (2)	
H26A	0.4245	0.4278	0.2496	0.128*	
H26B	0.5132	0.3837	0.2233	0.128*	
H26C	0.5703	0.4328	0.2685	0.128*	
C27	0.4662 (7)	0.3451 (2)	0.4156 (8)	0.087 (2)	
H27A	0.4620	0.3386	0.5005	0.131*	
H27B	0.5345	0.3271	0.3810	0.131*	
H27C	0.3884	0.3355	0.3782	0.131*	
N31	1.3593 (4)	0.6433 (2)	0.0561 (4)	0.0711 (15)	
H31A	1.3637	0.6341	0.1302	0.085*	
C31	1.3821 (5)	0.6886 (2)	0.0291 (5)	0.0633 (14)	
H31	1.4006	0.7105	0.0903	0.076*	
C32	1.3792 (5)	0.70398 (18)	−0.0866 (4)	0.0502 (11)	
H32	1.3973	0.7361	−0.1052	0.060*	
C33	1.3490 (4)	0.67117 (15)	−0.1779 (4)	0.0405 (9)	
C34	1.3235 (5)	0.62408 (17)	−0.1419 (5)	0.0545 (12)	
H34	1.3016	0.6008	−0.1990	0.065*	
C35	1.3302 (5)	0.6120 (2)	−0.0266 (6)	0.0681 (16)	
H35	1.3135	0.5801	−0.0043	0.082*	
N32	1.3453 (4)	0.68469 (15)	−0.2895 (3)	0.0501 (9)	
C36	1.3831 (7)	0.7329 (2)	−0.3241 (5)	0.0726 (17)	
H36A	1.3123	0.7545	−0.3178	0.109*	
H36B	1.4128	0.7326	−0.4057	0.109*	
H36C	1.4494	0.7439	−0.2718	0.109*	
C37	1.3129 (6)	0.6522 (2)	−0.3847 (5)	0.0686 (16)	
H37A	1.3423	0.6202	−0.3653	0.103*	
H37B	1.3516	0.6629	−0.4581	0.103*	
H37C	1.2230	0.6516	−0.3947	0.103*	
N41	0.8097 (4)	0.38604 (16)	0.4387 (4)	0.0545 (10)	
H41A	0.8123	0.3756	0.3656	0.065*	
C41	0.8374 (5)	0.4314 (2)	0.4627 (4)	0.0547 (12)	
H41	0.8600	0.4519	0.3999	0.066*	
C42	0.8341 (5)	0.44863 (17)	0.5732 (4)	0.0487 (11)	
H42	0.8533	0.4811	0.5871	0.058*	
C43	0.8021 (4)	0.41871 (15)	0.6694 (4)	0.0397 (9)	
C44	0.7732 (4)	0.37120 (16)	0.6395 (4)	0.0460 (10)	
H44	0.7502	0.3493	0.6993	0.055*	
C45	0.7785 (4)	0.35695 (18)	0.5256 (5)	0.0521 (11)	
H45	0.7590	0.3249	0.5072	0.063*	
N42	0.7971 (3)	0.43422 (13)	0.7805 (3)	0.0422 (8)	
C46	0.8383 (5)	0.48182 (18)	0.8139 (5)	0.0551 (12)	
H46A	0.9288	0.4828	0.8144	0.083*	
H46B	0.8073	0.4895	0.8928	0.083*	
H46C	0.8066	0.5050	0.7570	0.083*	
C47	0.7646 (5)	0.40293 (18)	0.8784 (4)	0.0555 (12)	
H47A	0.6905	0.3847	0.8579	0.083*	
H47B	0.7485	0.4220	0.9489	0.083*	
H47C	0.8331	0.3811	0.8940	0.083*	
N51	0.6896 (4)	0.64325 (18)	1.0088 (4)	0.0593 (11)	
H51A	0.6857	0.6310	0.9375	0.071*	
C51	0.7084 (5)	0.6894 (2)	1.0227 (4)	0.0569 (13)	
H51	0.7190	0.7086	0.9546	0.068*	
C52	0.7132 (5)	0.71033 (17)	1.1305 (4)	0.0490 (11)	
H52	0.7275	0.7435	1.1372	0.059*	
C53	0.6965 (4)	0.68204 (16)	1.2337 (4)	0.0410 (9)	
C54	0.6802 (4)	0.63271 (16)	1.2136 (5)	0.0486 (11)	
H54	0.6718	0.6119	1.2790	0.058*	
C55	0.6763 (5)	0.61493 (19)	1.1044 (5)	0.0554 (12)	
H55	0.6640	0.5818	1.0937	0.066*	
N52	0.6956 (4)	0.70091 (14)	1.3407 (3)	0.0456 (8)	
C56	0.7191 (6)	0.75123 (19)	1.3597 (5)	0.0598 (13)	
H56A	0.7894	0.7612	1.3114	0.090*	
H56B	0.7380	0.7567	1.4432	0.090*	
H56C	0.6458	0.7696	1.3374	0.090*	
C57	0.6639 (6)	0.6725 (2)	1.4439 (4)	0.0602 (14)	
H57A	0.5838	0.6572	1.4312	0.090*	
H57B	0.6593	0.6931	1.5134	0.090*	
H57C	0.7273	0.6482	1.4565	0.090*	
N61	1.0625 (5)	0.7312 (2)	1.1934 (5)	0.0788 (16)	
H61A	1.0731	0.7508	1.2531	0.095*	
C61	1.0528 (6)	0.7490 (2)	1.0834 (5)	0.0693 (16)	
H61	1.0596	0.7823	1.0710	0.083*	
C62	1.0336 (5)	0.71981 (18)	0.9909 (4)	0.0531 (12)	
H62	1.0273	0.7325	0.9135	0.064*	
C63	1.0228 (4)	0.67040 (16)	1.0088 (4)	0.0405 (9)	
C64	1.0364 (5)	0.6541 (2)	1.1262 (4)	0.0565 (12)	
H64	1.0310	0.6211	1.1427	0.068*	
C65	1.0566 (6)	0.6847 (3)	1.2137 (4)	0.0682 (16)	
H65	1.0669	0.6732	1.2918	0.082*	
N62	0.9989 (4)	0.64083 (14)	0.9185 (4)	0.0494 (9)	
C66	0.9958 (6)	0.6572 (2)	0.7978 (4)	0.0632 (14)	
H66A	0.9126	0.6687	0.7788	0.095*	
H66B	1.0174	0.6310	0.7449	0.095*	
H66C	1.0552	0.6832	0.7879	0.095*	
C67	0.9799 (6)	0.59025 (19)	0.9366 (6)	0.0702 (15)	
H67A	1.0601	0.5742	0.9413	0.105*	
H67B	0.9324	0.5772	0.8705	0.105*	
H67C	0.9345	0.5852	1.0102	0.105*	

### Tris[4-(dimethylamino)pyridinium] pentakis(thiocyanato-κN)manganate(II) (Compound2) Atomic displacement parameters (Å^2^)

**Table:**

	*U*^11^	*U*^22^	*U*^33^	*U*^12^	*U*^13^	*U*^23^
Mn1	0.0469 (4)	0.0414 (3)	0.0345 (3)	−0.0005 (3)	−0.0011 (3)	−0.0013 (3)
N1	0.067 (3)	0.046 (2)	0.043 (2)	0.0004 (19)	0.0006 (18)	−0.0003 (16)
C1	0.046 (2)	0.044 (2)	0.034 (2)	0.0030 (19)	−0.0026 (17)	−0.0016 (17)
S1	0.0689 (8)	0.0426 (6)	0.0648 (8)	−0.0032 (6)	−0.0125 (6)	−0.0045 (6)
N2	0.065 (3)	0.073 (3)	0.053 (2)	0.017 (2)	−0.018 (2)	−0.016 (2)
C2	0.054 (3)	0.045 (3)	0.038 (2)	0.007 (2)	−0.002 (2)	−0.0031 (18)
S2	0.0493 (7)	0.0650 (8)	0.0456 (6)	0.0016 (5)	−0.0038 (5)	0.0087 (5)
N3	0.056 (3)	0.070 (3)	0.049 (2)	0.000 (2)	−0.0003 (19)	−0.015 (2)
C3	0.046 (2)	0.052 (2)	0.036 (2)	−0.003 (2)	0.0021 (18)	0.0002 (19)
S3	0.0702 (9)	0.0738 (9)	0.0470 (7)	0.0103 (7)	0.0079 (6)	−0.0144 (6)
N4	0.058 (3)	0.063 (3)	0.048 (2)	−0.008 (2)	0.0065 (19)	0.0034 (19)
C4	0.051 (3)	0.046 (2)	0.041 (2)	−0.004 (2)	0.000 (2)	−0.0018 (19)
S4	0.0985 (11)	0.0599 (8)	0.0480 (7)	−0.0245 (8)	0.0151 (7)	0.0002 (6)
N5	0.054 (2)	0.053 (2)	0.045 (2)	−0.0017 (19)	−0.0054 (18)	0.0007 (17)
C5	0.051 (3)	0.044 (2)	0.035 (2)	−0.0018 (19)	0.0022 (19)	−0.0019 (17)
S5	0.0549 (8)	0.0944 (12)	0.0588 (8)	0.0104 (7)	−0.0132 (6)	−0.0238 (7)
Mn2	0.0451 (4)	0.0395 (3)	0.0385 (3)	−0.0017 (3)	0.0036 (3)	0.0007 (3)
N6	0.061 (3)	0.067 (3)	0.059 (3)	−0.005 (2)	−0.005 (2)	−0.003 (2)
C6	0.057 (3)	0.056 (3)	0.040 (2)	−0.002 (2)	−0.005 (2)	0.002 (2)
S6	0.1235 (16)	0.0809 (11)	0.0533 (8)	−0.0148 (10)	−0.0296 (9)	−0.0062 (7)
N7	0.051 (2)	0.051 (2)	0.054 (2)	−0.0028 (19)	0.0101 (18)	0.0062 (18)
C7	0.050 (2)	0.040 (2)	0.039 (2)	−0.0039 (19)	0.0021 (18)	0.0033 (18)
S7	0.0526 (7)	0.0610 (7)	0.0479 (6)	−0.0016 (5)	0.0104 (5)	−0.0098 (5)
N8	0.062 (3)	0.046 (2)	0.048 (2)	−0.0048 (19)	0.0068 (19)	−0.0021 (18)
C8	0.044 (2)	0.043 (2)	0.036 (2)	0.0013 (18)	0.0044 (17)	−0.0020 (17)
S8	0.0638 (8)	0.0422 (6)	0.0661 (8)	−0.0086 (6)	0.0106 (6)	−0.0089 (5)
N9	0.059 (3)	0.058 (2)	0.050 (2)	0.008 (2)	0.002 (2)	0.003 (2)
C9	0.046 (2)	0.041 (2)	0.041 (2)	−0.0019 (19)	0.0022 (19)	−0.0005 (18)
S9	0.0634 (8)	0.0674 (8)	0.0474 (6)	0.0149 (6)	−0.0055 (6)	0.0034 (6)
N10	0.055 (2)	0.058 (2)	0.057 (2)	−0.004 (2)	0.0150 (19)	−0.002 (2)
C10	0.054 (3)	0.042 (2)	0.038 (2)	−0.003 (2)	0.0042 (18)	0.0003 (18)
S10	0.0489 (6)	0.0768 (8)	0.0497 (6)	0.0023 (6)	0.0081 (5)	0.0151 (6)
N11	0.054 (2)	0.072 (3)	0.045 (2)	0.013 (2)	0.0027 (18)	0.013 (2)
C11	0.062 (3)	0.070 (3)	0.043 (3)	0.011 (3)	−0.005 (2)	−0.013 (2)
C12	0.063 (3)	0.043 (2)	0.046 (3)	0.005 (2)	−0.002 (2)	−0.0074 (19)
C13	0.042 (2)	0.042 (2)	0.038 (2)	0.0014 (18)	0.0009 (18)	−0.0025 (17)
C14	0.048 (3)	0.041 (2)	0.049 (3)	−0.0019 (19)	0.002 (2)	0.0000 (19)
C15	0.050 (3)	0.051 (3)	0.062 (3)	0.001 (2)	−0.001 (2)	0.012 (2)
N12	0.061 (3)	0.047 (2)	0.0382 (19)	−0.0014 (18)	0.0054 (17)	−0.0016 (16)
C16	0.082 (4)	0.057 (3)	0.056 (3)	−0.007 (3)	0.004 (3)	0.015 (2)
C17	0.085 (4)	0.073 (4)	0.040 (2)	−0.004 (3)	−0.001 (3)	−0.009 (2)
N21	0.070 (3)	0.076 (3)	0.060 (3)	0.015 (3)	−0.007 (2)	−0.030 (2)
C21	0.071 (4)	0.058 (3)	0.079 (4)	0.002 (3)	−0.015 (3)	−0.008 (3)
C22	0.067 (3)	0.055 (3)	0.047 (3)	−0.002 (2)	−0.006 (2)	0.005 (2)
C23	0.042 (2)	0.051 (3)	0.037 (2)	0.0027 (19)	−0.0066 (18)	−0.0073 (18)
C24	0.054 (3)	0.066 (3)	0.047 (3)	0.007 (2)	0.002 (2)	0.009 (2)
C25	0.065 (3)	0.102 (5)	0.032 (2)	0.018 (3)	−0.002 (2)	−0.005 (3)
N22	0.065 (3)	0.061 (3)	0.053 (2)	0.009 (2)	−0.010 (2)	−0.020 (2)
C26	0.089 (5)	0.128 (6)	0.039 (3)	0.018 (4)	−0.001 (3)	−0.029 (3)
C27	0.078 (4)	0.059 (4)	0.125 (6)	0.009 (3)	−0.029 (4)	−0.033 (4)
N31	0.053 (3)	0.108 (4)	0.052 (3)	0.016 (3)	0.012 (2)	0.031 (3)
C31	0.059 (3)	0.090 (4)	0.041 (3)	0.007 (3)	−0.001 (2)	−0.006 (3)
C32	0.061 (3)	0.049 (3)	0.041 (2)	0.002 (2)	−0.005 (2)	−0.006 (2)
C33	0.038 (2)	0.042 (2)	0.041 (2)	0.0004 (17)	0.0016 (18)	−0.0019 (17)
C34	0.055 (3)	0.040 (2)	0.068 (3)	−0.001 (2)	0.014 (2)	−0.003 (2)
C35	0.058 (3)	0.064 (3)	0.082 (4)	0.012 (3)	0.020 (3)	0.027 (3)
N32	0.052 (2)	0.060 (2)	0.0378 (19)	−0.0007 (19)	−0.0016 (17)	−0.0042 (17)
C36	0.093 (5)	0.071 (4)	0.053 (3)	−0.004 (3)	0.003 (3)	0.020 (3)
C37	0.066 (4)	0.093 (4)	0.047 (3)	−0.003 (3)	−0.005 (3)	−0.022 (3)
N41	0.051 (2)	0.068 (3)	0.044 (2)	0.006 (2)	−0.0032 (18)	−0.0072 (19)
C41	0.054 (3)	0.069 (3)	0.041 (2)	0.000 (2)	−0.001 (2)	0.007 (2)
C42	0.050 (3)	0.045 (2)	0.051 (3)	−0.003 (2)	0.002 (2)	0.008 (2)
C43	0.034 (2)	0.041 (2)	0.044 (2)	0.0022 (17)	0.0007 (17)	0.0040 (18)
C44	0.047 (3)	0.041 (2)	0.050 (2)	0.0006 (19)	−0.004 (2)	0.0024 (19)
C45	0.043 (2)	0.051 (3)	0.062 (3)	0.007 (2)	−0.005 (2)	−0.008 (2)
N42	0.045 (2)	0.0417 (19)	0.0402 (19)	−0.0029 (16)	0.0031 (15)	0.0020 (15)
C46	0.063 (3)	0.052 (3)	0.050 (3)	−0.011 (2)	0.002 (2)	−0.003 (2)
C47	0.065 (3)	0.054 (3)	0.047 (3)	0.000 (2)	0.013 (2)	0.008 (2)
N51	0.050 (2)	0.078 (3)	0.050 (2)	0.010 (2)	0.0034 (19)	−0.014 (2)
C51	0.057 (3)	0.071 (3)	0.043 (3)	0.012 (3)	0.002 (2)	0.009 (2)
C52	0.052 (3)	0.048 (2)	0.047 (3)	0.005 (2)	0.001 (2)	0.010 (2)
C53	0.034 (2)	0.043 (2)	0.045 (2)	0.0005 (17)	−0.0001 (17)	0.0049 (18)
C54	0.044 (2)	0.041 (2)	0.061 (3)	0.0004 (19)	0.003 (2)	0.003 (2)
C55	0.051 (3)	0.052 (3)	0.064 (3)	0.000 (2)	0.005 (2)	−0.012 (2)
N52	0.050 (2)	0.047 (2)	0.0399 (19)	−0.0011 (17)	−0.0011 (16)	0.0052 (16)
C56	0.068 (3)	0.048 (3)	0.063 (3)	−0.002 (2)	−0.012 (3)	−0.003 (2)
C57	0.069 (4)	0.070 (3)	0.041 (3)	−0.009 (3)	0.003 (2)	0.010 (2)
N61	0.090 (4)	0.090 (4)	0.056 (3)	−0.015 (3)	0.006 (3)	−0.030 (3)
C61	0.091 (4)	0.057 (3)	0.060 (3)	−0.016 (3)	0.017 (3)	−0.014 (3)
C62	0.066 (3)	0.046 (3)	0.047 (3)	−0.001 (2)	0.013 (2)	−0.003 (2)
C63	0.042 (2)	0.045 (2)	0.035 (2)	0.0045 (18)	0.0013 (17)	−0.0019 (17)
C64	0.062 (3)	0.065 (3)	0.043 (3)	0.008 (2)	0.005 (2)	0.009 (2)
C65	0.078 (4)	0.095 (5)	0.032 (2)	0.013 (3)	0.000 (2)	−0.003 (3)
N62	0.054 (2)	0.046 (2)	0.048 (2)	0.0043 (17)	0.0004 (18)	−0.0066 (17)
C66	0.073 (4)	0.078 (4)	0.040 (3)	0.017 (3)	−0.008 (2)	−0.012 (2)
C67	0.081 (4)	0.044 (3)	0.086 (4)	−0.001 (3)	0.012 (3)	−0.010 (3)

### Tris[4-(dimethylamino)pyridinium] pentakis(thiocyanato-κN)manganate(II) (Compound2) Geometric parameters (Å, º)

**Table:**

Mn1—N4	2.099 (4)	C32—C33	1.425 (6)
Mn1—N1	2.104 (4)	C32—H32	0.9500
Mn1—N3	2.128 (4)	C33—N32	1.323 (6)
Mn1—N2	2.198 (5)	C33—C34	1.415 (7)
Mn1—N5	2.205 (4)	C34—C35	1.352 (8)
N1—C1	1.167 (6)	C34—H34	0.9500
C1—S1	1.611 (5)	C35—H35	0.9500
N2—C2	1.162 (6)	N32—C37	1.458 (6)
C2—S2	1.627 (5)	N32—C36	1.472 (7)
N3—C3	1.156 (6)	C36—H36A	0.9800
C3—S3	1.608 (5)	C36—H36B	0.9800
N4—C4	1.160 (6)	C36—H36C	0.9800
C4—S4	1.608 (5)	C37—H37A	0.9800
N5—C5	1.157 (6)	C37—H37B	0.9800
C5—S5	1.628 (5)	C37—H37C	0.9800
Mn2—N6	2.100 (5)	N41—C45	1.325 (7)
Mn2—N8	2.100 (4)	N41—C41	1.339 (7)
Mn2—N9	2.103 (4)	N41—H41A	0.8800
Mn2—N10	2.205 (4)	C41—C42	1.344 (7)
Mn2—N7	2.217 (4)	C41—H41	0.9500
N6—C6	1.158 (7)	C42—C43	1.421 (6)
C6—S6	1.605 (5)	C42—H42	0.9500
N7—C7	1.166 (6)	C43—N42	1.334 (6)
C7—S7	1.632 (4)	C43—C44	1.415 (6)
N8—C8	1.171 (6)	C44—C45	1.354 (7)
C8—S8	1.609 (5)	C44—H44	0.9500
N9—C9	1.163 (6)	C45—H45	0.9500
C9—S9	1.617 (5)	N42—C47	1.461 (6)
N10—C10	1.168 (6)	N42—C46	1.463 (6)
C10—S10	1.622 (5)	C46—H46A	0.9800
N11—C11	1.328 (7)	C46—H46B	0.9800
N11—C15	1.339 (7)	C46—H46C	0.9800
N11—H11A	0.8800	C47—H47A	0.9800
C11—C12	1.357 (7)	C47—H47B	0.9800
C11—H11	0.9500	C47—H47C	0.9800
C12—C13	1.424 (6)	N51—C51	1.325 (7)
C12—H12	0.9500	N51—C55	1.354 (7)
C13—N12	1.331 (6)	N51—H51A	0.8800
C13—C14	1.424 (6)	C51—C52	1.359 (7)
C14—C15	1.360 (7)	C51—H51	0.9500
C14—H14	0.9500	C52—C53	1.427 (6)
C15—H15	0.9500	C52—H52	0.9500
N12—C16	1.446 (7)	C53—N52	1.325 (6)
N12—C17	1.454 (6)	C53—C54	1.419 (6)
C16—H16A	0.9800	C54—C55	1.336 (7)
C16—H16B	0.9800	C54—H54	0.9500
C16—H16C	0.9800	C55—H55	0.9500
C17—H17A	0.9800	N52—C56	1.456 (6)
C17—H17B	0.9800	N52—C57	1.459 (6)
C17—H17C	0.9800	C56—H56A	0.9800
N21—C21	1.321 (8)	C56—H56B	0.9800
N21—C25	1.334 (9)	C56—H56C	0.9800
N21—H21A	0.8800	C57—H57A	0.9800
C21—C22	1.353 (8)	C57—H57B	0.9800
C21—H21	0.9500	C57—H57C	0.9800
C22—C23	1.404 (7)	N61—C65	1.332 (8)
C22—H22	0.9500	N61—C61	1.348 (8)
C23—N22	1.349 (6)	N61—H61A	0.8800
C23—C24	1.394 (7)	C61—C62	1.349 (7)
C24—C25	1.339 (8)	C61—H61	0.9500
C24—H24	0.9500	C62—C63	1.411 (7)
C25—H25	0.9500	C62—H62	0.9500
N22—C27	1.448 (8)	C63—N62	1.345 (6)
N22—C26	1.466 (8)	C63—C64	1.415 (6)
C26—H26A	0.9800	C64—C65	1.333 (8)
C26—H26B	0.9800	C64—H64	0.9500
C26—H26C	0.9800	C65—H65	0.9500
C27—H27A	0.9800	N62—C66	1.445 (7)
C27—H27B	0.9800	N62—C67	1.453 (7)
C27—H27C	0.9800	C66—H66A	0.9800
N31—C35	1.326 (9)	C66—H66B	0.9800
N31—C31	1.333 (8)	C66—H66C	0.9800
N31—H31A	0.8800	C67—H67A	0.9800
C31—C32	1.383 (7)	C67—H67B	0.9800
C31—H31	0.9500	C67—H67C	0.9800
			
N4—Mn1—N1	115.03 (17)	C35—C34—H34	119.9
N4—Mn1—N3	123.99 (19)	C33—C34—H34	119.9
N1—Mn1—N3	120.97 (18)	N31—C35—C34	121.9 (5)
N4—Mn1—N2	91.71 (19)	N31—C35—H35	119.0
N1—Mn1—N2	91.61 (18)	C34—C35—H35	119.0
N3—Mn1—N2	86.71 (17)	C33—N32—C37	122.3 (5)
N4—Mn1—N5	90.38 (17)	C33—N32—C36	120.8 (4)
N1—Mn1—N5	91.55 (17)	C37—N32—C36	116.7 (5)
N3—Mn1—N5	88.42 (17)	N32—C36—H36A	109.5
N2—Mn1—N5	175.07 (16)	N32—C36—H36B	109.5
C1—N1—Mn1	165.5 (4)	H36A—C36—H36B	109.5
N1—C1—S1	177.1 (4)	N32—C36—H36C	109.5
C2—N2—Mn1	172.6 (4)	H36A—C36—H36C	109.5
N2—C2—S2	179.2 (5)	H36B—C36—H36C	109.5
C3—N3—Mn1	168.4 (4)	N32—C37—H37A	109.5
N3—C3—S3	179.8 (6)	N32—C37—H37B	109.5
C4—N4—Mn1	178.2 (4)	H37A—C37—H37B	109.5
N4—C4—S4	179.4 (5)	N32—C37—H37C	109.5
C5—N5—Mn1	175.6 (4)	H37A—C37—H37C	109.5
N5—C5—S5	178.7 (4)	H37B—C37—H37C	109.5
N6—Mn2—N8	121.35 (19)	C45—N41—C41	119.7 (4)
N6—Mn2—N9	121.77 (19)	C45—N41—H41A	120.1
N8—Mn2—N9	116.88 (18)	C41—N41—H41A	120.1
N6—Mn2—N10	90.22 (18)	N41—C41—C42	121.9 (5)
N8—Mn2—N10	89.77 (17)	N41—C41—H41	119.1
N9—Mn2—N10	89.23 (18)	C42—C41—H41	119.1
N6—Mn2—N7	92.53 (18)	C41—C42—C43	120.5 (5)
N8—Mn2—N7	90.07 (16)	C41—C42—H42	119.8
N9—Mn2—N7	88.06 (17)	C43—C42—H42	119.8
N10—Mn2—N7	176.88 (18)	N42—C43—C44	121.8 (4)
C6—N6—Mn2	171.3 (5)	N42—C43—C42	122.7 (4)
N6—C6—S6	178.2 (5)	C44—C43—C42	115.5 (4)
C7—N7—Mn2	162.7 (4)	C45—C44—C43	120.0 (5)
N7—C7—S7	178.0 (5)	C45—C44—H44	120.0
C8—N8—Mn2	163.4 (4)	C43—C44—H44	120.0
N8—C8—S8	177.8 (4)	N41—C45—C44	122.5 (5)
C9—N9—Mn2	177.8 (4)	N41—C45—H45	118.8
N9—C9—S9	179.1 (5)	C44—C45—H45	118.8
C10—N10—Mn2	169.0 (4)	C43—N42—C47	122.0 (4)
N10—C10—S10	179.5 (5)	C43—N42—C46	122.1 (4)
C11—N11—C15	120.2 (4)	C47—N42—C46	115.4 (4)
C11—N11—H11A	119.9	N42—C46—H46A	109.5
C15—N11—H11A	119.9	N42—C46—H46B	109.5
N11—C11—C12	123.0 (5)	H46A—C46—H46B	109.5
N11—C11—H11	118.5	N42—C46—H46C	109.5
C12—C11—H11	118.5	H46A—C46—H46C	109.5
C11—C12—C13	118.9 (5)	H46B—C46—H46C	109.5
C11—C12—H12	120.6	N42—C47—H47A	109.5
C13—C12—H12	120.6	N42—C47—H47B	109.5
N12—C13—C14	121.7 (4)	H47A—C47—H47B	109.5
N12—C13—C12	121.8 (4)	N42—C47—H47C	109.5
C14—C13—C12	116.5 (4)	H47A—C47—H47C	109.5
C15—C14—C13	120.0 (4)	H47B—C47—H47C	109.5
C15—C14—H14	120.0	C51—N51—C55	120.0 (5)
C13—C14—H14	120.0	C51—N51—H51A	120.0
N11—C15—C14	121.4 (5)	C55—N51—H51A	120.0
N11—C15—H15	119.3	N51—C51—C52	122.5 (5)
C14—C15—H15	119.3	N51—C51—H51	118.7
C13—N12—C16	121.4 (4)	C52—C51—H51	118.7
C13—N12—C17	121.0 (4)	C51—C52—C53	119.4 (5)
C16—N12—C17	117.5 (4)	C51—C52—H52	120.3
N12—C16—H16A	109.5	C53—C52—H52	120.3
N12—C16—H16B	109.5	N52—C53—C54	122.6 (4)
H16A—C16—H16B	109.5	N52—C53—C52	121.9 (4)
N12—C16—H16C	109.5	C54—C53—C52	115.5 (4)
H16A—C16—H16C	109.5	C55—C54—C53	121.3 (5)
H16B—C16—H16C	109.5	C55—C54—H54	119.3
N12—C17—H17A	109.5	C53—C54—H54	119.3
N12—C17—H17B	109.5	C54—C55—N51	121.2 (5)
H17A—C17—H17B	109.5	C54—C55—H55	119.4
N12—C17—H17C	109.5	N51—C55—H55	119.4
H17A—C17—H17C	109.5	C53—N52—C56	121.6 (4)
H17B—C17—H17C	109.5	C53—N52—C57	121.2 (4)
C21—N21—C25	120.5 (5)	C56—N52—C57	117.1 (4)
C21—N21—H21A	119.7	N52—C56—H56A	109.5
C25—N21—H21A	119.7	N52—C56—H56B	109.5
N21—C21—C22	121.3 (6)	H56A—C56—H56B	109.5
N21—C21—H21	119.3	N52—C56—H56C	109.5
C22—C21—H21	119.3	H56A—C56—H56C	109.5
C21—C22—C23	119.6 (5)	H56B—C56—H56C	109.5
C21—C22—H22	120.2	N52—C57—H57A	109.5
C23—C22—H22	120.2	N52—C57—H57B	109.5
N22—C23—C24	122.6 (5)	H57A—C57—H57B	109.5
N22—C23—C22	120.5 (5)	N52—C57—H57C	109.5
C24—C23—C22	116.9 (4)	H57A—C57—H57C	109.5
C25—C24—C23	120.1 (5)	H57B—C57—H57C	109.5
C25—C24—H24	120.0	C65—N61—C61	121.5 (5)
C23—C24—H24	120.0	C65—N61—H61A	119.3
N21—C25—C24	121.5 (5)	C61—N61—H61A	119.3
N21—C25—H25	119.3	N61—C61—C62	120.3 (6)
C24—C25—H25	119.3	N61—C61—H61	119.9
C23—N22—C27	122.0 (5)	C62—C61—H61	119.9
C23—N22—C26	121.0 (5)	C61—C62—C63	120.2 (5)
C27—N22—C26	117.0 (5)	C61—C62—H62	119.9
N22—C26—H26A	109.5	C63—C62—H62	119.9
N22—C26—H26B	109.5	N62—C63—C62	121.1 (4)
H26A—C26—H26B	109.5	N62—C63—C64	122.3 (5)
N22—C26—H26C	109.5	C62—C63—C64	116.6 (5)
H26A—C26—H26C	109.5	C65—C64—C63	120.4 (5)
H26B—C26—H26C	109.5	C65—C64—H64	119.8
N22—C27—H27A	109.5	C63—C64—H64	119.8
N22—C27—H27B	109.5	N61—C65—C64	121.1 (5)
H27A—C27—H27B	109.5	N61—C65—H65	119.5
N22—C27—H27C	109.5	C64—C65—H65	119.5
H27A—C27—H27C	109.5	C63—N62—C66	121.8 (4)
H27B—C27—H27C	109.5	C63—N62—C67	121.8 (4)
C35—N31—C31	121.2 (5)	C66—N62—C67	116.4 (4)
C35—N31—H31A	119.4	N62—C66—H66A	109.5
C31—N31—H31A	119.4	N62—C66—H66B	109.5
N31—C31—C32	120.9 (5)	H66A—C66—H66B	109.5
N31—C31—H31	119.5	N62—C66—H66C	109.5
C32—C31—H31	119.5	H66A—C66—H66C	109.5
C31—C32—C33	119.4 (5)	H66B—C66—H66C	109.5
C31—C32—H32	120.3	N62—C67—H67A	109.5
C33—C32—H32	120.3	N62—C67—H67B	109.5
N32—C33—C34	122.7 (4)	H67A—C67—H67B	109.5
N32—C33—C32	121.0 (4)	N62—C67—H67C	109.5
C34—C33—C32	116.3 (4)	H67A—C67—H67C	109.5
C35—C34—C33	120.3 (5)	H67B—C67—H67C	109.5

### Tris[4-(dimethylamino)pyridinium] pentakis(thiocyanato-κN)manganate(II) (Compound2) Hydrogen-bond geometry (Å, º)

**Table:**

*D*—H···*A*	*D*—H	H···*A*	*D*···*A*	*D*—H···*A*
N11—H11*A*···S2	0.88	2.37	3.224 (4)	163
C11—H11···S9^i^	0.95	3.02	3.945 (5)	166
C15—H15···S8^ii^	0.95	2.86	3.728 (5)	153
C16—H16*B*···S9^iii^	0.98	3.02	3.954 (6)	160
C17—H17*B*···S9^iii^	0.98	2.96	3.930 (6)	170
N21—H21*A*···S1^iv^	0.88	2.82	3.520 (5)	138
N21—H21*A*···S7	0.88	2.81	3.485 (6)	134
C25—H25···N1^iv^	0.95	2.62	3.567 (7)	175
C26—H26*B*···N5	0.98	2.58	3.500 (7)	157
N31—H31*A*···S10	0.88	2.41	3.266 (5)	164
C31—H31···S3^v^	0.95	2.99	3.838 (6)	150
C35—H35···S1^vi^	0.95	2.96	3.512 (6)	118
C36—H36*B*···S3^vii^	0.98	2.99	3.868 (6)	149
N41—H41*A*···S5	0.88	2.45	3.302 (4)	163
C41—H41···S6	0.95	2.94	3.804 (5)	152
C45—H45···S8^viii^	0.95	2.88	3.445 (5)	119
C47—H47*C*···S2^ix^	0.98	2.98	3.717 (5)	133
N51—H51*A*···S7	0.88	2.43	3.288 (5)	163
C51—H51···S4^x^	0.95	2.99	3.931 (5)	169
C55—H55···S1^iv^	0.95	2.93	3.746 (5)	145
C57—H57*C*···N7^iv^	0.98	2.69	3.539 (7)	146
N61—H61*A*···S8^iv^	0.88	2.78	3.507 (5)	141
C65—H65···N8^iv^	0.95	2.66	3.513 (7)	150
C66—H66*A*···S4^x^	0.98	2.92	3.767 (6)	145

Symmetry codes: (i) *x*−1, *y*, *z*−1; (ii) −*x*+1, *y*−1/2, −*z*; (iii) *x*−1, *y*, *z*−2; (iv) *x*, *y*, *z*+1; (v) −*x*+2, *y*+1/2, −*z*; (vi) *x*+1, *y*, *z*; (vii) −*x*+2, *y*+1/2, −*z*−1; (viii) −*x*+2, *y*−1/2, −*z*+1; (ix) *x*+1, *y*, *z*+1; (x) −*x*+1, *y*+1/2, −*z*+1.
